# Opportunities and Challenges of a Cap-and-Trade System for Plastics

**DOI:** 10.1021/acs.est.4c04931

**Published:** 2025-01-24

**Authors:** Hadeel Al-Zawaidah, Marlene Kammerer, Denise M. Mitrano, Kryss Waldschläger

**Affiliations:** † Wageningen University and Research, Hydrology and Environmental Hydraulics Group, 6700 AA Wageningen, The Netherlands; ‡ Oeschger Centre for Climate Change Research, Institute for Political Science, University of Bern, Fabrikstrasse 8, 3012 Bern, Switzerland; § Department Environmental Social Sciences, eawag Aquatic Research, Überlandstrasse 133, 8600 Dübendorf, Switzerland; ⊥ Environmental Systems Science Department, ETH Zurich, Universitätsstrasse 16, 8092 Zurich, Switzerland

**Keywords:** market-based instruments, plastics policy, cap-and-trade system, circular economy, global governance

## Abstract

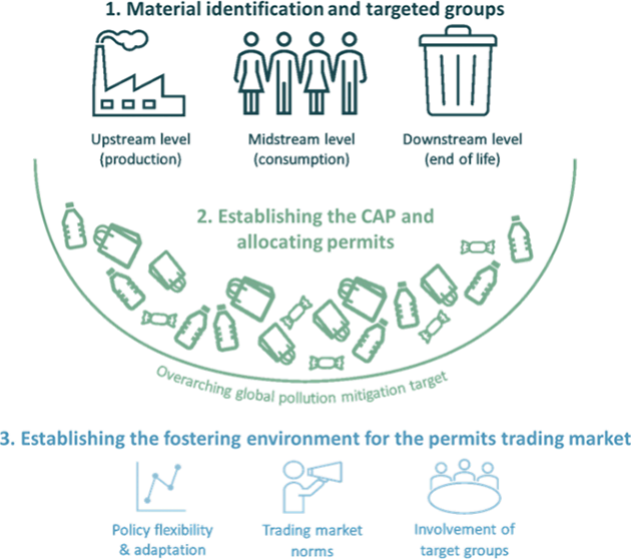

Recently, the rapid increase in global plastics production has caused various ecological and economic issues, worsened by poor material and waste management. Among the market-based instruments that could help mitigate the environmental impacts of plastics throughout their life-cycle, we evaluate the advantages and limitations of incorporating a cap-and-trade (CAT) system into future policy mixes. Our aim is to inspire further investigation of CAT’s feasibility rather than presenting it as the ultimate solution. Drawing from past CAT implementations in domains such as water resource management and carbon emissions, we outline three key policy design considerations: (1) material and target group identification, (2) cap establishment and permit allocation, and (3) development of a competitive market environment. We explore a three-tiered approach with global, national, and sectoral caps covering the plastic lifecycle from cradle to grave. While there are viable reasons to consider a plastics CAT, significant challenges persist, which may ultimately limit its implementation. In the context of ongoing UN Plastics Treaty negotiations or future policy developments, this evaluation of CAT can be beneficial for assessing when and how this tool can address the negative externalities of plastics.

## Abstract 2

Growing global plastics production and waste management challenges initiated the UN Plastics Treaty negotiations. This manuscript explores the integration of a cap-and-trade system to manage plastics, highlighting advantages and limitations.

## 1 Introduction

Plastics have long been indispensable in global material production due to their versatility and cost-effectiveness.^1−3^ In 2018, the global plastics trade reached USD 1 trillion, representing 5% of total global trade.^4^ Since the 1950s, plastics production has grown exponentially, currently reaching an annual output of 430 million metric tons and employing approximately nine million people worldwide.^5^ However, the prevalent linear single-use model and inadequate waste management practices have led to ecological and economic challenges surpassing planetary boundaries.^6,7^ Environmental consequences include feedstock acquisition,^8,9^ greenhouse gas (GHG) emissions, and plastics pollution.^10,11^ Due, in part, to reliance on coal combustion,^6^ plastics production alone could contribute up to 13% of gross carbon emissions by 2050.^12^ In addition to unwanted plastics waste emissions into the environment, insufficient recycling due to polymer mixes, additive use, degradation, and nonpolymeric contamination further exacerbate material losses,^13^ resulting in annual losses of USD 80–120 billion worth of plastics.^14^

In 2022, the *United Nations Environmental Program* (UNEP) established an International Negotiation Committee tasked with developing an international legally binding agreement on plastics pollution (hereafter, UN Plastics Treaty) by the end of 2024.^15^ The prospective treaty aims to combine nationally and internationally determined approaches, providing flexibility in instrument selection at the national level to address plastics pollution across the materials’ life-cycle.^16^

To date, policymakers have often chosen policy instruments that aim to eradicate or reduce the use of specific products or materials.^17^ Typically, bans are the instrument type of choice (e.g., a ban on plastic bags or other single-use items, such as straws). Also popular are levies on specific products (e.g., plastic bags) to disincentivize their use. While bans or levies can be effective for a specific product, they are often criticized for being ineffective in reducing the overall demand for virgin plastics^18^ while increasing the demand for other alternatives (often not more sustainable ones).^19^ Meanwhile, more integrative methods that pursue a life-cycle or circular economy approach, such as extended producer responsibility, are complex and build on voluntary agreements or cooperation. They are criticized for a lack of enforcement mechanisms and high costs.^20,21^ Therefore, the individual approaches currently employed cannot be considered a viable solution for plastics, and many researchers and practitioners call for a “holistic” approach that builds on a mix of policy instruments targeting different plastics around their life-cycle,^22,23^ while at same time not creating an overcomplex regulatory landscape.^24^

Against this background, this study examines the potential of a market-based instrument, namely, a comprehensive cap-and-trade (CAT) system, to regulate different types of plastics and products under one regulatory framework. We do not intend to present such a CAT system as the ultimate policy solution but as a potential instrument in the policy mix. We assess the applicability of a CAT system tailored to the specificities of plastics, drawing on the experience with CAT systems developed over the past three decades that have addressed complex environmental challenges. Additionally, we discuss the strengths and limitations of implementing such a CAT system for plastics. Our objective is to initiate a dialogue and lay the groundwork for further discussions and refinements among academics and policymakers working toward a production, consumption, and disposal of plastics with a reduced environmental footprint.

## 2 Material and Methods

### 2.1 Brief History of International Plastics Pollution Policies

The rising awareness of the problems related to the excessive production, consumption, and disposal of plastics is linked to an increasing number of policies adopted worldwide.^17,25−27^ The design of these plastics policies has primarily been driven by regional, national, or local contexts, depending on the policy at hand. This has resulted in a fragmented policy landscape that often does not account for the global scale of the issue. While some international agreements related to plastics pollution exist, there is currently no comprehensive international policy framework that contains binding commitments to tackle the regulation of plastics across the materials’ entire value chain.^17,25,28−30^

Historically, international regulations regarding plastics as environmental pollutants were aimed at controlling and preventing marine pollution. The first international agreement that mentioned plastics as a potential pollutant was the 1972 *London Convention* (Convention on the Prevention of Marine Pollution by Dumping of Wastes and Other Matter). In 1973, the *International Convention for the Prevention of Pollution from Ships* (MARPOL) extended this regulation to ships, covering the disposal of all plastics at sea.^31^ Addressing land-based sources, the 1989 *Basel Convention* (on the Control of Transboundary Movements of Hazardous Wastes and Their Disposal) concretely addressed plastics waste as a hazardous material. The recent amendments in 2019 to Annexes II, VIII, and IX further refined the rules for specific plastic types, especially those that are challenging to recycle. These three conventions remain the only legally binding international agreements addressing plastics, primarily from a waste treatment perspective and thus only concerning their end of life.

Since then, several nonbinding international policies (international soft law), designed to assist governments in implementing effective policies and committing different target groups to voluntary action, have been introduced. Karasik et al.^25^ identified 28 international policies that have been introduced since 2000, with a primary focus on land-based plastics sources. These include resolutions, declarations, and decisions by various UN bodies, such as the *General Assembly*, *Environment Assembly* (UNEA), the *Convention on Biological Diversity*, the *Convention on Migratory Species*, and the UNEP, as well as action plans and charters by *G20* and *G7*.^17,25,27^

The most prominent international soft law to regulate plastics is the 2011 *Honolulu Strategy*. This policy document is a global framework developed by the UNEP and the *National Oceanic and Atmospheric Administration* aimed at reducing the scope and impact of plastics pollution. It was designed to provide policy ideas to governments, industry, nongovernmental organizations, and other target groups at all governmental levels (i.e., global, regional, national, and local).^32^ Similarly, guidelines on the monitoring and assessment of plastics in rivers and lakes^33^ and in the oceans^34^ are provided by UNEP and the *Joint Group of Experts on the Scientific Aspects of Marine Environmental Protection*. Such monitoring programs are essential to achieve the relevant targets of the Sustainable Development Goals on marine pollution.^35^ On March 2, 2022, decided, in a historic resolution (UNEA 5/14), to negotiate an international legally binding treaty to address plastics pollution. The future UN Plastics Treaty is expected to be implemented by the end of 2024 and is supposed to address the “full life-cycle of plastic from source to sea,” drawing on both binding and voluntary measures.^36^ Hence, a transformation of the plastics economy from linear to circular will be a key component of the new treaty (see^5^ for more details on potential transition pathways).

**Figure 1:**
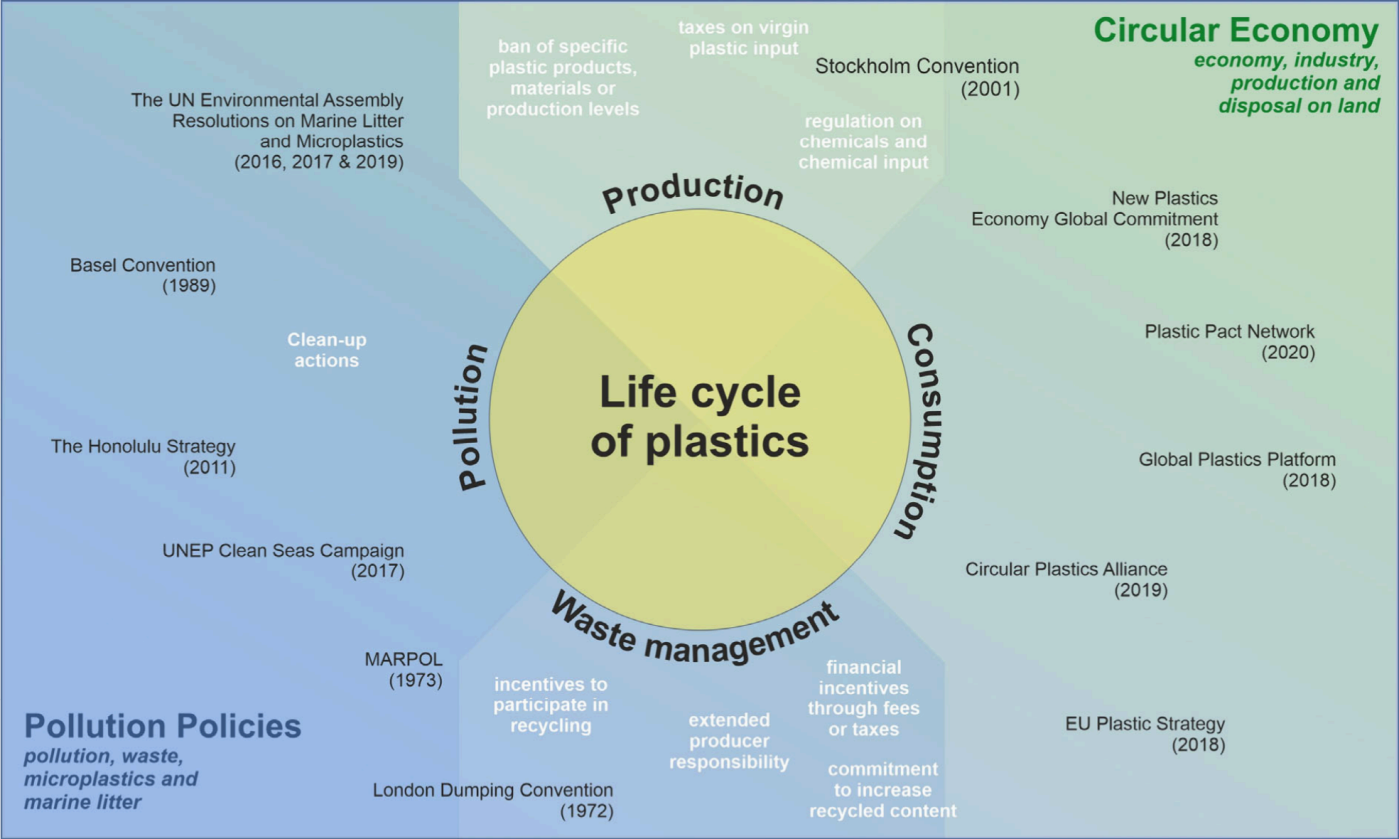
Life-cycle of plastics in combination with the two main trends in global plastics governance (according to Nielsen et al.^37^): potential actions (white text) and policy regulations (black text).

While numerous other international agreements tend to focus on plastics at the end of their life-cycle, including reduced pollution, marine litter, and waste management, more recent international initiatives promote a circular economy approach that explicitly includes production and consumption, as well (Figure 1). In this context, the proposed UN Plastics Treaty is part of a rather new trend in international policy to assess broader materials’ use cycles under one umbrella.

### 2.2 Design Features of Cat Systems to Govern Complex Environmental Problems

Market-based instruments (MBIs), such as levies, taxes, or GHG emission trading, are widely perceived as effective tools for regulating environmental issues by stimulating technological, social, or economic changes. Change is thereby induced by setting market incentives rather than imposing bans or standards. While bans and standards aim to avoid polluting behavior completely or focus on the pollution levels of one specific product or process, the target of MBIs is to readjust pollution levels where it is most cost-effective for the target group and to align resource consumption or pollution with sustainability goals. Several economists and policymakers have advocated for MBIs in different sectors since the 1990s,^38−40^ as they offer several potential advantages, including (1) flexibility for target groups by aligning their interests with environmental goals, (2) technological advancement through financial incentives, and (3) achievement of environmental protection cost-efficiently.^41^ Conversely, other stakeholders challenge or even oppose MBIs because they perceive their implementation of putting a price on environmental health and safety as morally and ethically objectionable. In any case, in practice, MBI implementation is often confronted with political obstacles. For example, it can be challenging to set the optimal level of a tax or levy due to political opposition or a lack of information. As a result, taxes or levies are often too lax and thus do not yield the desired results. Furthermore, MBIs often face resistance from producers and consumers alike, since they increase the costs of a specific product and may endanger market competitiveness^41^ in particular when environmental costs are not equalized across the members of a governance system. Thus, market participants in the stricter regulatory areas will be confronted by a competitive disadvantage compared to those in less-regulated areas. This leaves the consumers with more expensive options in the local markets.^41^

**Figure 2:**
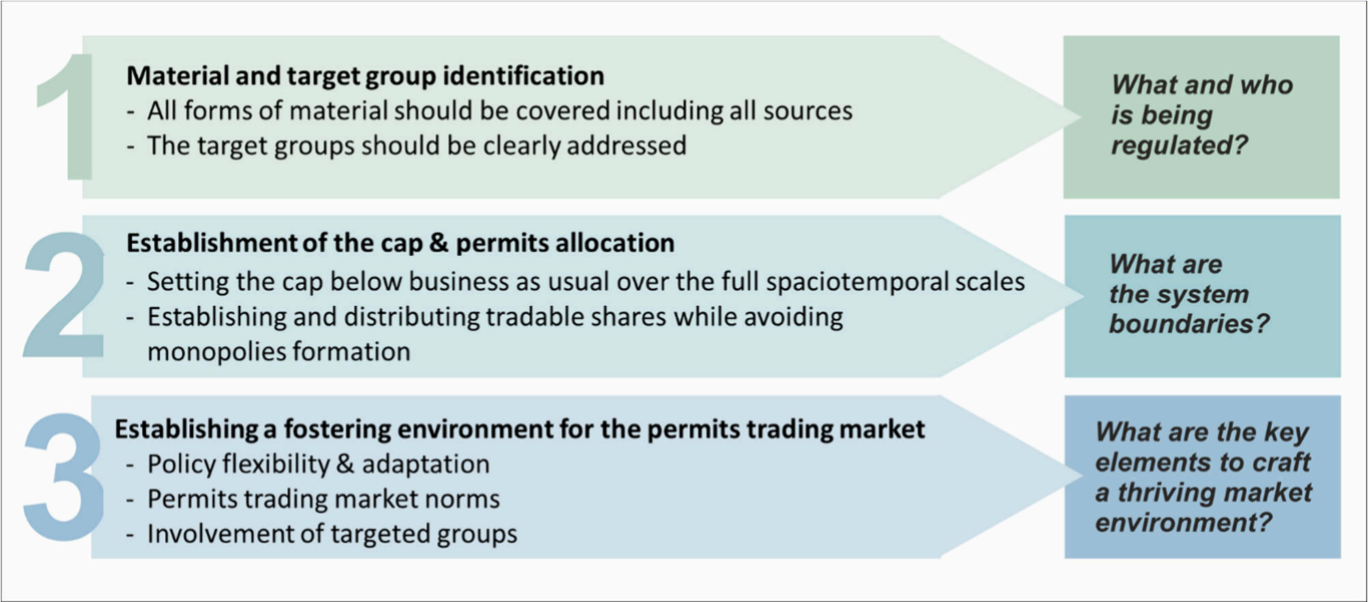
Summary of policy design requirements for CAT systems, based on Table S2, which provides further details and examples.

Prominent MBIs are cap-and-trade systems that aim to induce change by creating an artificial market for a specific pollutant or resource. One prominent example of such a CAT system is the emissions trading system of the European Union (EU ETS), where emissions are capped to a certain level, certificates issues, and then traded.^42^ CAT systems are complex governance arrangements involving diverse public and private target groups, often across territorial or political boundaries.^42−44^ Past experiences in various domains, including air pollution, fisheries, water pollutants, and water resource management (see Table S1), have shown that tailoring CAT systems to specific contexts is crucial, as there is no universal solution fitting the realities of every pollutant or resource. Indeed, factors influencing system design and applicability vary significantly, and not all environmental issues suit CAT regulations.^36,45,46^ However, three core elements can be deduced from past experiences,^42,43,47^ as displayed in Figure 2 and explained in detail below.

First, the resources or pollutants to be regulated, along with the relevant target groups, must be identified. This process requires accounting for an often heterogeneous mix of pollutants or resources, as observed during the development of water pollution and mixed-stock fisheries CAT systems.^48,49^ Second, a cap needs to be set and a permit allocation system needs to be established. For this, a regulating body (e.g., a governmental agency) defines the maximum allowable level of the pollutant (e.g., GHG emissions emitted in a country) or resource use (e.g., water consumption) under the cap. Setting a cap that covers the entire spatiotemporal scale of the regulated resource is crucial to preventing leakage. For example, the limited geographic coverage of the Greenhouse Gas Initiative in the United States led to industry migration outside the regulated region.^50^ The cap must also be set below business-as-usual (BAU) levels, as demonstrated by the EU Emissions Trading System, where setting a cap above BAU levels caused market collapse.^51^ The cap is then divided into smaller, tradable permits with terms carefully selected to prevent monopolies and foster a dynamic market. This was critical in the San Joaquin Basin water CAT system, where inadequate permit ownership led to reduced market competitiveness.^52^ Third, a CAT policy needs to define norms and rules to establish an effective market environment. Here, three elements are key: defining trading market norms, involving target groups, and ensuring policy flexibility. Clear market norms, as seen in the Acid Rain Program to reduce SO_2_ and NO_*x*_ emissions in the United States, are essential for creating a dynamic market and reducing uncertainty.^53^ Involving all target groups from the early design phases can prevent conflicts and enhance compliance, as demonstrated by the Lead Scheme and Pacific Habitat in Alaska fisheries.^54,55^ Finally, CAT systems are volatile and require flexibility to adapt to changing political and economic landscapes. The water CAT system in China and the Carbon Emissions Program in Tokyo highlight the importance of continuous feedback and policy adaptability for broader applicability and success.^56,57^

## 3 Results and Discussion

In this section, we explore whether CAT could be an integrative component of the future UN Plastics Treaty or future policy mixes by addressing the design features outlined above. We conclude by evaluating the advantages and limitations of CAT in mitigating the negative environmental impacts of plastic production, consumption, and disposal.

### 3.1 Material and Target Group Identification along the Life-Cycle of Plastics

Identifying the plastic materials covered by the CAT system is complex as the term “plastics” covers a diverse and complex mixture of polymers (e.g., virgin petroleum-based plastics, biobased plastics, biodegradable plastics, and recycled plastics) and additives that may experience a change in properties over time.^26^ Given that many products are actually a combination of polymers or multilayered materials, categorizing plastics in the CAT system by polymer type complicates the process of defining what the target material could or should be. Additionally, the environmental impact varies for different materials and products throughout their lifespan.^26^ Potential solutions for dealing with this diversity in plastic materials and its implications for establishing a cap are discussed in section 3.2. In addition to the covered material, target groups for the CAT system need to be identified. Potential target groups span various economic sectors and political levels along the full life-cycle,^36^ as outlined in the most recent UN Plastics Treaty draft.^16,58^ Therefore, tailored analyses of potential target groups are necessary for each country due to differences in plastics production, consumption, and disposal, and a phased approach, considering upstream (production), midstream (consumption), and downstream (waste) stages, might provide a holistic view of the plastic life cycle and relevant target groups. National authorities should conduct these analyses and report regularly to the Treaty’s management body. Clear guidelines and capacity-building efforts to develop these reports, especially for less developed countries, are crucial, as recognized by the UNEP.^5^

### 3.2 Establishing the Cap and Allocating Permits

**Figure 3:**
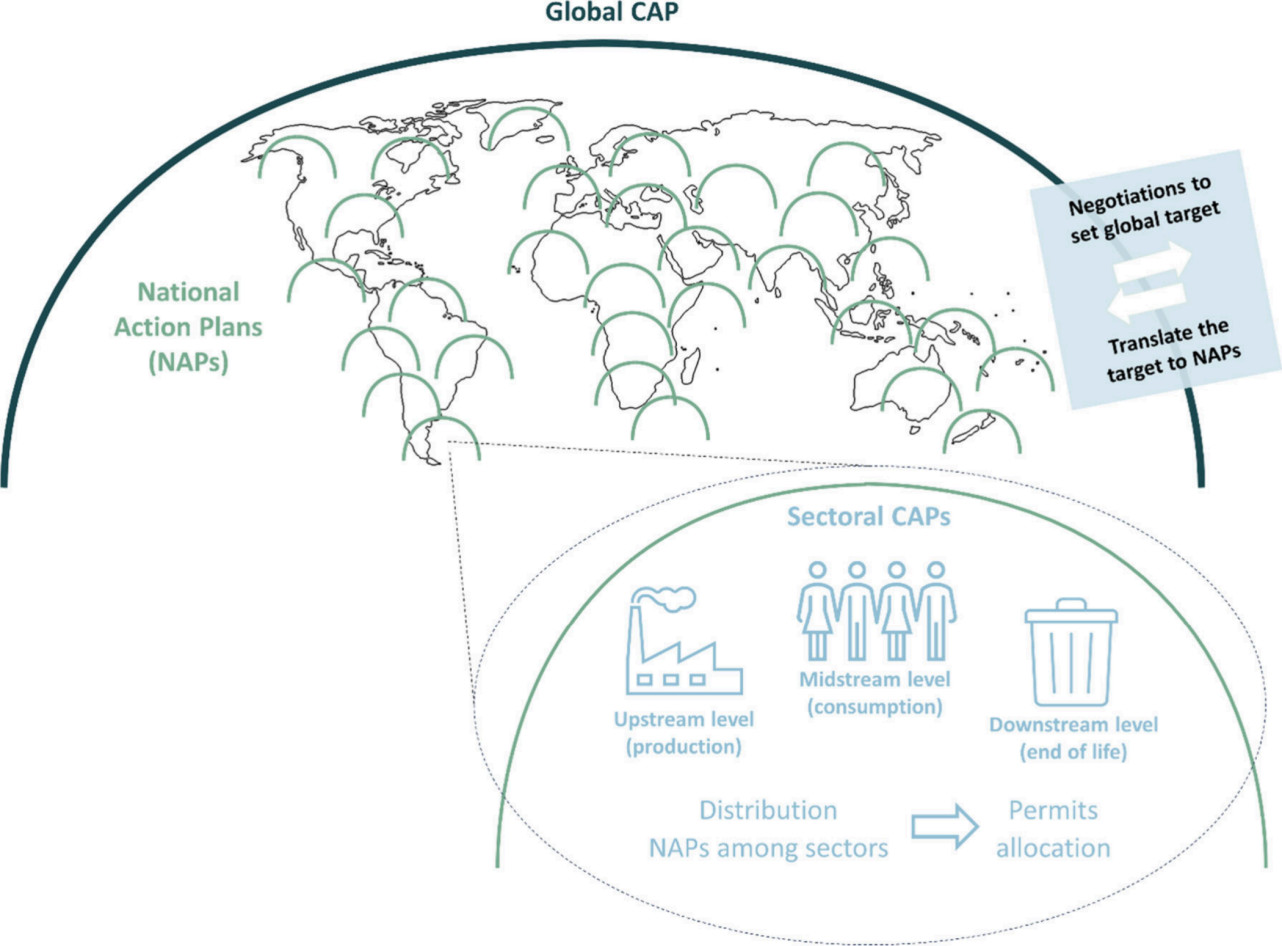
An envisioned three-level global plastics CAT system.

The central issue to address is how to integrate the complex plastics network, which encompasses multiple target groups and life-cycle phases, into a unified global system.^59^ To tackle this, we examined a three-level global CAT system (Figure 3).

The first level is typically a global cap. Analogous to the two degrees temperature goal under the Paris Agreement to mitigate climate change,^60^ such a cap would quantify the international mitigation target. To establish this cap, the international community must agree on sustainable production and recycling rates using criteria such as acceptable global concentrations of plastics in the environment (e.g., in soil, water, and air) and other externalities within plastics production (e.g., water use and GHG emissions), which are then translated into measurable units (e.g., tons or items per cubic meter). However, setting these levels is challenging, particularly due to the lack of a global consensus on the associated risks (defined as exposure time hazard), which can vary based on plastic composition, item or particle size, and other factors.

To ensure universal participation in such a global CAT system, countries must be granted flexibility in adopting it at the national level, considering different countries’ needs and capacities.^46,51,59^ Hence, the second level comprises country-specific caps formulated and regularly updated by the parties contributing to the UN Plastics Treaty, similar to the Paris Agreement’s ratcheting-up mechanism. Each country could submit the nationally self-determined caps regularly (e.g., every second year), which can be part of the National Action Plans (NAP) requested under the plastics treaty,^58^ detailing current national pollution levels, reductions, the national cap, regulated target groups, and materials, as well as the policies and measures required to integrate CAT into the respective national-level legislation. The cap should be progressively stringent, with each successive limit set lower than the previously approved cap, to achieve incremental reductions in pollution levels. A key factor for success at this level is the ability to track plastics across the life cycle and national boundaries. Addressing transboundary plastics pollution presents significant challenges, as observed with other pollutants and resources.^61−63^ Strategic behavior often undermines collective efforts, with countries prioritizing short-term economic gains over long-term environmental goals. This can manifest as underreporting pollution levels, avoiding stringent caps, or relying on other nations to shoulder mitigation responsibilities.^64^ Moreover, legal frameworks frequently lag behind, focusing on consultation and information-sharing rather than enforceable liability or substantive controls.^65^ The transboundary transport of pollutants also highlights the need for policies that account for geographical and economic asymmetries between countries.^66^

Recommendations to address these challenges have been explored^65^ and could be adapted for implementing caps at the national level. However, plastics pose additional complexities as they are often produced in one country, used in another, and discarded in a third. This necessitates robust tracking systems. A potential solution is a paper trail (e.g., import-export documents) to monitor plastics flows and shift permits across borders. However, the practicality of this approach remains uncertain due to the significant bureaucracy involved and the varying capacities of countries to prevent illegal activities.

The third level comprises a translation of national caps into smaller, tradable permits for different national trading markets, covering the identified target groups and materials across the full life-cycle of plastics. Covering plastics’ diversity while maintaining a manageable number of permits presents a challenge far beyond past CAT experiences.^67^ Addressing the permits challenge through past experiences with GHG emissions^68^ has yielded two options. The first is to translate all plastics and polymer types into a common metric that captures their capacity for environmental effects. However, establishing a unified metric across the entire value chain of the plastic life-cycle that captures and quantifies the diverse effects on humans, fauna, and ecosystems is challenging and requires further research. The second, and perhaps more feasible, option is to use a simple metric unit (e.g., kilograms) for plastics permits, regardless of the material’s composition. While this approach simplifies implementation, it risks overlooking the chemical and physical diversities of plastics. Ultimately, selecting the unit of plastics permits is a balancing act between reflecting the diversity of plastic materials and ensuring that the number of permits does not undermine market competition. A potential starting point could be the introduction of metric-unit permits, which could later be refined with a set of multipliers that account for material characteristics such as recyclability, lifespan, and potential for leakage and hazard. As an example, extended producer responsibility schemes have already proposed a recyclability index (RI) for plastics credits to address material diversity.^67^ However, relying on the RI alone might overshadow other important factors, such as the lifespan of the plastics product. For example, although polyethylene terephthalate (PET) has a high recyclability potential, a high proportion of single-use materials are made from PET (e.g., packaging), which may more readily be leaked to the environment.^1^ In contrast, products made out of poly(vinyl chloride) are often used for applications with longer lifespans (e.g., buildings and cars) and are thus less likely to end up in the environment. Additionally, RI is not suitable to address all plastic materials such as biodegradable plastics.^69−71^ Thus, the development of appropriate multipliers for the metric-unit would need to occur gradually, informed by comprehensive research and emerging insights on plastics.

Permit allocation methods could include auctioning or free allocation based on criteria such as the grandparenting approach, sector benchmarking, and output-based allocation.^46^ The selected allocation approach should be further examined in pilot studies, considering market dynamics, such as new company formations, mergers, and closures. Balancing competing sector interests at the permit level is crucial,^55^ with a focus on preventing permit concentration (monopoly formation) to maintain market competition.^52^

### 3.3 Establishing a Fostering Environment for the Market

A fostering permits trading market environment relies on three aspects: involving target groups, defining trading market norms, and ensuring policy flexibility. When developing policies for plastics, note that different target groups within this arena (e.g., plastics producers, product designers, product generators, material recovery facilities, recyclers, municipalities, and consumers) are affected to varying degrees.^72^ The risk of local target groups steering the market away from the overarching goal in favor of personal interests must be restricted through clear rules and norms.

Overall, market norms should ideally maintain a streamlined design, minimize bureaucratic complexities, and diminish uncertainties. The World Bank’s Handbook of Emission Trading Schemes Design^46^ offers valuable recommendations in this regard (especially sections 4–7). While many of these lessons could also be applicable to the certificate trading market for the plastics CAT in principle—because of the complexity and heterogeneity of the plastics themselves—in addition to their global reach at different stages of the life-cycle—market norms are expected to be more challenging to optimize than in previous iterations of CAT systems. In this context, the goal of trading plastic permits is not to maximize profits but to secure the necessary permits to continue production. Balancing supply and demand fluctuations is key to prevent financial shocks, where price spikes and market collapse are avoided.^73^ Involving financial support entities (e.g., banks) can provide liquidity and facilitate additional market performance information^46^ by managing price and volume risks through specific fiscal actions.^46^ Price callers who combine an allowance reserve with the auction price floor should be considered for improved stability and investment planning. In extreme cases, where economic imperatives challenge environmental goals, market regulators should be empowered to intervene through intentional purchases. This offers an additional buffer in cases where the cap is not tight enough or in cases where urgent action is needed^74^

Globally, such a CAT system would entail integrating a monitoring body under the future UN Plastics Treaty umbrella and national bodies that report to the UN, as developing a market with various target groups requires an authoritative body to oversee standards. National parties, who will define caps, allocate permits, and monitor their trade, would then be obligated to regularly submit updated NDCs and reports to this global authority, enhancing transparency and providing data on market performance, changes in BAU conditions, and environmental impacts. The latter may include carbon emissions, leakages, and the associated impacts and the product consumption duration, which can then be translated into quantifiable goals (e.g., plastics concentration in the environment) and used for improving the permit unit, as discussed above.

Three common phrases can guide the development of the plastics CAT system over time: “learning by doing”, “communication is key”, and “bend but don’t break”. In a globalized world with dynamic political and economic uncertainties, crafting a perfect policy from scratch is nearly impossible.^75^ Internal learning, to refine policy opinions, facilitates a testing and evaluation phase in which decision-making is deliberately and continuously supported by an improved knowledge base.^57,76^ This should include time and funding allocation to investigate and determine the optimal market design features. Continuous feedback channels are crucial for effective policy adaptation and precautionary measures.^57^ This includes the above-mentioned reporting program as well as communication channels at the policy–science interface (e.g., on plastics impacts and pathways within the environment and the capacity to improve material design and recyclability).^57^ This would allow for scientific evidence to be translated into action through policy changes, ultimately leading to improved market performance. Finally, the CAT system needs to be flexible to deal with local contexts and uncertainties. Regarding local contexts, market standards should, for example, consider the sustainability of small market participants.^77^ The uncertainties encompassing plastics production, consumption, disposal demand, production, and long-term development cannot be completely avoided and should thus be accounted for. Current recycling capabilities and unavoidable leakage to the environment can also not be overlooked.^78^ Addressing the spatial limitations of policies for plastics, especially concerning diverse pollutant sources and resource forms, can reduce these leakage risks. Furthermore, integrating the plastics permits trading market with existing CAT, such as those for water resources and carbon emissions, can effectively reduce emissions throughout the supply chain.^78,79^ Since fossil fuels are currently the predominant feedstock for plastics production, a clear connection with CAT regulations on GHG emissions can be drawn. However, currently, plastics production can be seen as a loophole for the oil industry, as subsidies are still granted to produce plastics^28^ even though they contribute to GHG emissions. In addition, the manufacturing, distribution, collection, and recycling processes in the plastics industry are associated with significant energy consumption.^80^ Along the production chain, plastics production is also a significant consumer of water resources.^81,82^ Early acknowledgment of these connections in policy development will ultimately enhance overall market performance and addressing issues such as allowance overestimation and market volatility^51^ might improve the performance of the CAT policy used to combat climate change and water resource issues.

### 3.4 On the Feasibility of a Plastics CAT

**Figure 4:**
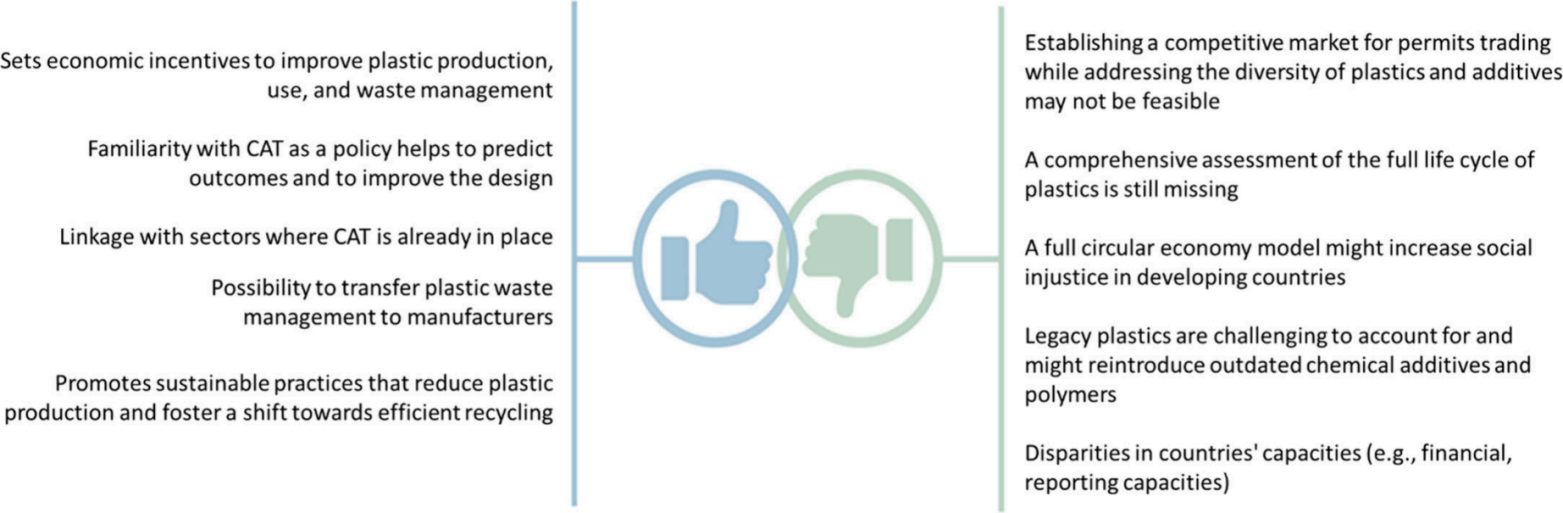
Overview of the potentials and limitations of plastics CAT.

Adopting a CAT system for plastics around their life-cycle fosters multiple opportunities (Figure 4). Familiarity with CAT as a tool to regulate resource use or pollution can reduce the difficulties in familiarizing the target groups involved in the policy and its functionalities, resulting in smoother adoption of the policy into the plastics sector. Additionally, over the past 30 years, considerable experience has been gained regarding the advantages and limitations of CAT policies for various applications.

To begin with, a CAT system facilitates the introduction of different permits covering various spatial and temporal scales of the plastics life-cycle and geographical distribution. This inclusive approach promotes sustainable practices, mitigating the devaluation of plastic products along their life-cycle and fostering a shift toward efficient recycling within a circular economy. Furthermore, the cap would limit and regulate the quantity and properties of the plastics allowed, making plastics scarcer. This scarcity would shift the cost–benefit ratio of plastics compared to alternative materials, encouraging their selection for specific applications based on a more comprehensive analysis. Hence, the economic incentives induced by CAT, along with the ability to raise the value of plastics near the end of their life-cycle (waste management), position it as a transformative force in reshaping existing plastic policies and driving sustainability across the entire life-cycle of plastics.

However, the introduction of such a comprehensive CAT system faces several challenges. A successful CAT system typically requires a competitive market characterized by a homogeneous, well-defined product and a large number of participants.^51,83^ The unprecedented diversity of polymers and additives used in plastics, varying across spatial and temporal scales as well as different economy models (circular versus linear), results in a heterogeneous product under the global cap. A potential alternative is to focus the CAT system on specific phases of the life cycle, as has recently been proposed for the production phase of primary plastics.^79^ However, while this approach might be effective for countries with active plastic production, it raises concerns for nations with no production but significant levels of plastic waste. Complementary policies would likely still be necessary to manage end-of-life plastics. Furthermore, the structure of the plastics across the life-cycle creates complex relationships among target groups, such as permit transfer between primary and secondary manufacturers and across (inter)national scales, which demands extensive regulations, complex design, and comprehensive documentation. Addressing these heterogeneities could lead to a proliferation of permits, resulting in a complex system and reduced participation for each permit type, thereby diminishing market competitiveness. In section 3.2, we presented different options to create a reasonably homogeneous product across national borders under the plastic cap, each with its own shortcomings. We conclude that there is currently no fully developed and immediately implementable solution to address the heterogeneities in creating a homogeneous product across national borders under the plastics cap. Without such a solution, the feasibility of implementing a comprehensive plastics cap-and-trade (CAT) system remains uncertain..

In addition, various socioeconomic and political realities create further limitations. For example, a growing interest in recycling can be anticipated under the CAT system, creating new job opportunities.^84^ However, variations in working conditions and technology use on a global scale could be concerning. Manual waste collection is more common in developing countries, often undertaken by unemployed individuals,^85^ potentially exacerbating social and environmental injustices linked to CAT policies.^86,87^ Furthermore, the feasibility of CAT is intertwined with the global political economy, involving the definition of global and national caps through negotiations akin to international agreements, such as climate negotiations. Despite efforts to raise awareness about plastics’ drawbacks, the availability of easier common practices might impede market shifts. Additionally, there may be potential economic disadvantages of compliance (e.g., reduced production revenue) in the short term, reducing the likelihood of full participation.

Most importantly, the success of a CAT system on a global scale depends on the willingness of the UN Plastics Treaty parties to agree on ambitious targets and formulate a respective goal. In the absence of sanction mechanisms—which means that countries cannot be forced to participate and comply and potential compromises may arise—the outcome of such negotiations is often the smallest common denominator, as the climate negotiations vividly show. As national caps and permit allocation become the subjects of public and political debate, political and economic pressures may hinder efforts to establish adequate and effective caps and determine the appropriate number of permits, potentially leading to limited success or policies with loopholes. In some countries, financial and capacity constraints, as well as corruption concerns, might hinder the implementation of a plastics CAT. Disparities in countries’ capacities, especially in estimating pollution levels and reporting, highlight challenges in the preparation and accuracy of NDCs. As mentioned before, these local conditions need to be balanced by financial and capacity building, which would require, for example, a common fund based on the allocation of resources from wealthier countries.

## 4 Final Remarks

Plastics are a crucial part of the global economy, offering material solutions in various sectors. However, concerns about resource consumption and environmental impacts throughout the plastics life cycle have become more acute. Managing plastics across the full life-cycle globally is challenging, and while several national policies exist, a global agreement to tackle these challenges is required to be effective on a larger scale. This is where the UN Plastics Treaty steps in, which has been under negotiation since 2022 and is currently being drafted. However, the search for an appropriate policy mix to achieve the UN Plastics Treaty’s goals is ongoing. Here, we looked into the possibility of implementing a CAT system to manage the plastics over the full life-cycle globally, addressing the pressing concerns surrounding resource utilization and environmental impacts throughout the plastics life-cycle. However, we acknowledge the complexity of this endeavor and the need for careful consideration of key design requirements for a plastic CAT scheme. Building on lessons learned from CAT schemes in other areas and the characteristics of plastics, we discussed three key design requirements for a plastics CAT scheme: (1) identification of plastics materials, (2) establishment of the cap and permit allocation, and (3) considerations to establish the trading market norms.

Our examination of the applicability of a CAT system to the complexities of plastics production, consumption, and disposal revealed multiple opportunities; however, critical challenges remain. CAT systems have shown promising results in mitigating other environmental concerns (e.g., air pollution, carbon emissions, water allocation, and fisheries) and offer a market-based approach to incentivizing sustainable practices across the plastics life-cycle. By setting caps on the various polluting factors of plastics production, use, and end of life and allowing for the trading of permits, CAT systems can align economic incentives with environmental objectives. Ultimately, this could help foster a transition to a reduction in primary plastics production and a circular plastics economy. However, the diverse material properties and applications of plastics and the international supply chain present hurdles. The array of polymers, additives, and different life-cycles for different products and applications complicates the establishment of clear regulations and permit frameworks. Moreover, social, political, and economic realities, including disparities in recycling infrastructure and global political dynamics, introduce additional layers of complexity.

As a market-based instrument that has been used before to curb resource use and mitigate pollution, a CAT system for plastics has the potential to decrease primary plastics production, incentivize recycling, and minimize environmental leakage. However, due to the various complexities attached to plastics (e.g., a diversity of materials, products, and applications and waste management strategies), a ready-to-implement plastics CAT system covering the full life-cycle is not currently within reach. This paper is meant as a foundation for stimulating discussions on how a future CAT system could be constructed. While a fully realized CAT system may take time, exploring CAT on a smaller scale (e.g., for a specific phase of the plastics life-cycle or at a local scale) and investigating complementary policies that could help address the limitations of a CAT system may yield valuable insights and practical solutions.

## Supporting Information Available

Summary of the lessons learned from different cap-and-trade systems in different sectors and summary of the lessons learned from different cap and trade systems in different sectors, summarized based on the three key design features (PDF)

## References

[ref1] Geyer R.; Jambeck J. R.; Law K. L. Production, Use, and Fate of All Plastics Ever Made. Sci. Adv. 2017, 3 (7), 25–29. 10.1126/sciadv.1700782. PMC5517107 28776036

[ref2] Villarrubia-Gómez P.; Almroth B. C.; Eriksen M.; Ryberg M.; Cornell S. E. Plastics Pollution and the Planetary Boundaries Framework. SSRN Journal 2022, 10.2139/ssrn.4254033.

[ref3] Bachmann M.; Zibunas C.; Hartmann J.; Tulus V.; Suh S.; Guillén-Gosálbez G.; Bardow A. Towards Circular Plastics within Planetary Boundaries. Nat. Sustain 2023, 6 (5), 599–610. 10.1038/s41893-022-01054-9.

[ref4] Barrowclough D.; Birkbeck C. D.; Christen J. Global Trade in Plastics: Insights from the First Life-Cycle Trade Database. In United Nations Conference on Trade and Development; UN, 2020.

[ref5] UNEP. Turning off the Tap How the World Can End Plastic Pollution and Create a Circular Economy, 2023. https://www.unep.org/resources/turning-off-tap-end-plastic-pollution-create-circular-economy (accessed 2023–07–23).

[ref6] Cabernard L.; Pfister S.; Oberschelp C.; Hellweg S. Growing Environmental Footprint of Plastics Driven by Coal Combustion. Nat. Sustain 2022, 5 (2), 139–148. 10.1038/s41893-021-00807-2.

[ref7] Bachmann M.; Zibunas C.; Hartmann J.; Tulus V.; Suh S.; Guillén-Gosálbez G.; Bardow A. Towards Circular Plastics within Planetary Boundaries. Nat. Sustain 2023, 6 (5), 599–610. 10.1038/s41893-022-01054-9.

[ref8] Tilsted J. P.; Bauer F.; Deere Birkbeck C.; Skovgaard J.; Rootzén J. Ending Fossil-Based Growth: Confronting the Political Economy of Petrochemical Plastics. One Earth 2023, 6 (6), 607–619. 10.1016/j.oneear.2023.05.018.

[ref9] Mitrano D. M.; Wagner M. A Sustainable Future for Plastics Considering Material Safety and Preserved Value. Nat. Rev. Mater. 2022, 7 (2), 71–73. 10.1038/s41578-021-00406-9.

[ref10] Law K. L.; Rochman C. M. Collaborations Uncover of Plastic Pollution. Nature 2023, 619, 254–255. 10.1038/d41586-023-02175-7. 37438596

[ref11] Mitrano D. M.; Wick P.; Nowack B. Placing Nanoplastics in the Context of Global Plastic Pollution. Nature Nanotechnology 2021, 491–500. 10.1038/s41565-021-00888-2. 33927363

[ref12] Hamilton L. A.; Feit S. Plastic & Climate: The Hidden Costs of a Plastic Planet; Washington, DC, 2019.

[ref13] Pivnenko K.; Jakobsen L. G.; Eriksen M. K.; Damgaard A.; Astrup T. F. Challenges in Plastics Recycling. In Proceedings of the Fifteenth Waste Management and Landfill Symposium; CISA Publisher: Sardinia, Italy, 2015.

[ref14] World Economic Forum; Ellen MacArthur Foundation; McKinsey & Company. New Plastics Economy—Rethinking the Future of Plastics, 2016. http://www.ellenmacarthurfoundation.org/publications (accessed 2023–08–12).

[ref15] UNEP. End Plastic Pollution: Towards an International Legally Binding Instrument; 2022.

[ref16] United Nations. Zero Draft Text of the International Legally Binding on Plastic Pollution, Including in the Marine; 2023.

[ref17] Diana Z.; Vegh T.; Karasik R.; Bering J.; D. Llano Caldas J.; Pickle A.; Rittschof D.; Lau W.; Virdin J. The Evolving Global Plastics Policy Landscape: An Inventory and Effectiveness Review. Environ. Sci. Policy 2022, 134, 34–45. 10.1016/j.envsci.2022.03.028.

[ref18] Herberz T.; Barlow C. Y.; Finkbeiner M. Sustainability Assessment of a Single-Use Plastics Ban. Sustainability (Switzerland) 2020, 12 (9), 3746 10.3390/su12093746.

[ref19] Convery F.; McDonnell S.; Ferreira S. The Most Popular Tax in Europe? Lessons from the Irish Plastic Bags Levy. Environ. Resour Econ (Dordr) 2007, 38 (1), 1–11. 10.1007/s10640-006-9059-2.

[ref20] Sicotte D. M.; Seamon J. L. Solving the Plastics Problem: Moving the U.S. from Recycling to Reduction. Soc. Nat. Resour 2021, 34 (3), 393–402. 10.1080/08941920.2020.1801922.

[ref21] Ogunola O. S.; Onada O. A.; Falaye A. E. Mitigation Measures to Avert the Impacts of Plastics and Microplastics in the Marine Environment (a Review). Environmental Science and Pollution Research 2018, 9293–9310. 10.1007/s11356-018-1499-z. 29470754

[ref22] Diana Z.; Vegh T.; Karasik R.; Bering J.; D. Llano Caldas J.; Pickle A.; Rittschof D.; Lau W.; Virdin J. The Evolving Global Plastics Policy Landscape: An Inventory and Effectiveness Review. Environ. Sci. Policy 2022, 134, 34–45. 10.1016/j.envsci.2022.03.028.

[ref23] Vince J.; Hardesty B. D. Plastic Pollution Challenges in Marine and Coastal Environments: From Local to Global Governance. Restor Ecol 2017, 25 (1), 123–128. 10.1111/rec.12388.

[ref24] Steensgaard I.; Syberg K.; Rist S.; Hartmann N.; Boldrin A.; Hansen S. F. From Macro- to Microplastics - Analysis of EU Regulation along the Life Cycle of Plastic Bags. Environ. Pollut. 2017, 289–299. 10.1016/j.envpol.2017.02.007. 28222979

[ref25] Karasik R.; Bering J.; Griffin M.; Diana Z.; Laspada C.; Schachter J.; Wang Y.; Pickle A.; Virdin J.; Affiliations A. Annual Trends in Plastics Policy: A Brief; Nicholas Institute, Duke University, 2022.

[ref26] Groh K. J.; Arp H. P. H.; MacLeod M.; Wang Z. Assessing and Managing Environmental Hazards of Polymers: Historical Development, Science Advances and Policy Options. Environ. Sci. Process Impacts 2023, 25 (1), 10–25. 10.1039/D2EM00386D. 36511246

[ref27] Duke University. Plastics Policy Inventory. https://nicholasinstitute.duke.edu/plastics-policy-inventory/search (accessed 2023–08–30).

[ref28] Borrelle S. B.; Rochman C. M.; Liboiron M.; Bond A. L.; Lusher A.; Bradshaw H.; Provencher J. F. Opinion Why We Need an International Agreement on Marine Plastic Pollution. Proceedings of the National Academy of Sciences 2017, 114, 9994–9997. 10.1073/pnas.1714450114. PMC5617320 28928233

[ref29] Raubenheimer K.; McIlgorm A. Is the Montreal Protocol a Model That Can Help Solve the Global Marine Plastic Debris Problem?. Mar Policy 2017, 81 (April), 322–329. 10.1016/j.marpol.2017.04.014.

[ref30] Dauvergne P. The Power of Environmental Norms: Marine Plastic Pollution and the Politics of Microbeads Pollution and the Politics of Microbeads. Env Polit 2018, 27 (4), 579–597. 10.1080/09644016.2018.1449090.

[ref31] International Maritime Organization. Articles of the International Convention for the Prevention of Pollution from Ships Amended by Resolution MEPC.111(50) Amended by Resolution MEPC.115(51) Amended by Resolution MEPC.116(51), 2005. http://www.mar.ist.utl.pt/mventura/Projecto-Navios-I/IMO-Conventions%20%28copies%29/MARPOL.pdf (accessed 2023–08–14).

[ref32] UNEP; NOAA. Honolulu Strategy A Global Framework for Prevention and Management of Marine Debris, 2011. https://wedocs.unep.org/20.500.11822/10670 (accessed 2023–08–15).

[ref33] UNEP. Water Pollution by Plastics and Microplastics: A Review of Technical Solutions from Source to Sea; 2020.

[ref34] GESAMP. Guidelines for the Monitoring and Assessment of Plastic Litter in the Ocean, 2019. http://gesamp.org.

[ref35] Agamuthu P.; Mehran S. B.; Norkhairah A.; Norkhairiyah A. Marine Debris: A Review of Impacts and Global Initiatives. Waste Management and Research 2019, 37 (10), 987–1002. 10.1177/0734242X19845041. 31084415

[ref36] UNEP. What you need to know about the plastic pollution resolution. https://www.unep.org/news-and-stories/story/what-you-need-know-about-plastic-pollution-resolution (accessed 2023–08–14).

[ref37] Nielsen T. D.; Hasselbalch J.; Holmberg K.; Stripple J. Politics and the Plastic Crisis: A Review throughout the Plastic Life Cycle. Wiley Interdiscip Rev. Energy Environ 2020, 9 (1), e360 10.1002/wene.360.

[ref38] Hahn R. W.; Stavins R. N. Incentive Based Environmental Regulation: A New Era from an Old Idea?. Ecol Law Q 1991, 18 (1), 1–42.

[ref39] Rosegrant M. W.; Binswanger H. P. Markets in Tradable Water Rights: Potential for Efficiency Gains in Developing Country Water Resource Allocation. World Dev 1994, 22 (11), 1613–1625. 10.1016/0305-750X(94)00075-1.

[ref40] Tietenberg T. H. Economic Instruments for Conservation. Oxf Rev. Econ Policy 1990, 6 (1), 17–33. 10.1093/oxrep/6.1.17.

[ref41] Stavins R. N. Experience with Market-Based Environmental Policy Instruments. In Handbook of Environmental Economics; Elsevier, 2003; Vol. 1, pp 355–435. 10.1016/S1574-0099(03)01014-3.

[ref42] Betz R.; Michaelowa A.; Castro P.; Kotsch R.; Mehling M.; Michaelowa K.; Baranzini A. The Carbon Market Challenge; Cambridge University Press, 2022. 10.1017/9781009216500.

[ref43] Connor J. D.; Bryan B. A.; Nolan M. Cap and Trade Policy for Managing Water Competition from Potential Future Carbon Plantations. Environ. Sci. Policy 2016, 66, 11–22. 10.1016/j.envsci.2016.07.005.

[ref44] Tietenberg T. Emissions Trading: An Exercise in Reforming Pollution Policy; Resources for the Future: Washington, D.C, 1985.

[ref45] Iftekhar S.; Fogarty J. Benefits of a Groundwater Allocation Trading Arrangement in a Water-Stressed Environment. Agric Water Manag 2022, 269, 107649 10.1016/j.agwat.2022.107649.

[ref46] PMR; ICAP. Emissions Trading in Practice: A Handbook on Design and Implementation, 2016. www.worldbank.org.

[ref47] Thompson C. L.; Supalla R. J.; Martin D. L.; McMullen B. P. Evidence Supporting Cap and Trade as a Groundwater Policy Option for Reducing Irrigation Consumptive Use. JAWRA Journal of the American Water Resources Association 2009, 45 (6), 1508–1518. 10.1111/j.1752-1688.2009.00384.x.

[ref48] Collentine D. Composite Market Design for a Transferable Discharge Permit (TDP) System. Journal of Environmental Planning and Management 2006, 49 (6), 929–946. 10.1080/09640560600947055.

[ref49] Karp W. A.; Melnychuk M. C.; Forrest R. E.; Little L. R.; McQuaw K.; Demarest C.; Hilborn R.; Baker N.; Mose B.; Turris B.; Lado E. P. Quota Use in Mixed-Stock Fisheries. Fish and Fisheries 2024, 25 (2), 251–267. 10.1111/faf.12806.

[ref50] Yan J. The Impact of Climate Policy on Fossil Fuel Consumption: Evidence from the Regional Greenhouse Gas Initiative (RGGI). Energy Econ 2021, 100, 105333 10.1016/j.eneco.2021.105333.

[ref51] Schmalensee R.; Stavins R. N. Lessons Learned from Three Decades of Experience with Cap and Trade. Rev. Environ. Econ Policy 2017, 11 (1), 59–79. 10.1093/reep/rew017.

[ref52] Young T. F.; Karkoski J. Green Evolution Are Economic Incentives the next Step in Nonpoint Source Pollution Control. Water Policy 2000, 2 (3), 151–173. 10.1016/S1366-7017(00)00006-4.

[ref53] Borghesi S. Water Tradable Permits: A Review of Theoretical and Case Studies. Journal of Environmental Planning and Management 2014, 57 (9), 1305–1332. 10.1080/09640568.2013.820175.

[ref54] Hahn R. W. Economic Prescriptions for Environmental Problems: How the Patient Followed the Doctor’s Orders. Journal of Economic Perspective 1989, 3 (2), 95–114. 10.1257/jep.3.2.95.

[ref55] Harrison H. L. Managing Many Nets: Possible Scenarios and Impacts for the Expansion of Cook Inlet Personal Use Fisheries. Fish Res. 2021, 236, 105811 10.1016/j.fishres.2020.105811.

[ref56] Moore S. M. The Development of Water Markets in China: Progress, Peril, and Prospects. Water Policy 2015, 17 (2), 253–267. 10.2166/wp.2014.063.

[ref57] Heggelund G.; Stensdal I.; Duan M. China’s Carbon Market: Potential for Success?. Politics and Governance 2022, 10 (1), 265–274. 10.17645/pag.v10i1.4792.

[ref58] Intergovernmental Negotiating Committee. Draft Text of the International Legally Binding Instrument on Plastic Pollution, Including in the Marine Environment; United Nations, 2024.

[ref59] Wang C.; Sun W.; Lim M. K.; Hu X.; Gao Y.; Ghadimi P. Structural Evolution of Global Plastic Life Cycle Trade: A Multilayer Network Perspective. Sustain Prod Consum 2022, 33, 1031–1042. 10.1016/j.spc.2022.08.027.

[ref60] UNFCCC. The Paris Agreement; UNFCCC: New York, 2015.

[ref61] Just R. E.; Netanyahu S. Conflict and Cooperation on Trans-Boundary Water Resources; Springer Science & Business Media, 1998; Vol. 11.

[ref62] Wang X.; Wang Y.; Pang Y.; Wang K.; Yu J. Pollution Load Coordination and Eco-Compensation for Trans-Boundary Water Pollution Control: The Case of the Tri-Border Region of the Yangtze Delta. Sustainability (Switzerland) 2024, 16 (3), 1151 10.3390/su16031151.

[ref63] Duda A. M. Achieving Pollution Prevention Goals for Transboundary Waters through International Joint Commission Processes. Water Sci. Technol. 1994, 30 (5), 223–231. 10.2166/wst.1994.0241.

[ref64] Tahvonen O.; Kaitala V.; Pohjola M. A Finnish - Soviet Acid Rain Game: Noncooperative Equilibria, Cost Efficiency, and Sulfur Agreements. J. Environ. Econ Manage 1993, 24 (1), 87–100. 10.1006/jeem.1993.1006.

[ref65] Merrill T. W. Golden Rules for Transboundary Pollution. Duke Law J. 1997, 46 (5), 931–1011. 10.2307/1372915.

[ref66] de Frutos J.; Martín-Herrán G. Spatial Effects and Strategic Behavior in a Multiregional Transboundary Pollution Dynamic Game. J. Environ. Econ Manage 2019, 97, 182–207. 10.1016/j.jeem.2017.08.001.

[ref67] Zhang X.; Liu C.; Medda F. A Smart-Contract-Aided Plastic Credit Scheme. IEEE Syst. J. 2023, 17 (1), 1703–1713. 10.1109/JSYST.2022.3205266.

[ref68] Monast J. J.; Virdin J. Pricing Plastics Pollution: Lessons from Three Decades of Pricing Climate Policy. Connecticut Law Review 2022, 54 (2), 345–390.

[ref69] Dilshad E.; Waheed H.; Ali U.; Amin A.; Ahmed I. General Structure and Classification of Bioplastics and Biodegradable Plastics. In Bioplastics for Sustainable Development; Springer: Singapore, 2021; pp 61–82.

[ref70] Mo A.; Zhang Y.; Gao W.; Jiang J.; He D. Environmental Fate and Impacts of Biodegradable Plastics in Agricultural Soil Ecosystems. Applied Soil Ecology 2023, 181, 104667 10.1016/j.apsoil.2022.104667.

[ref71] Tian L.; Jinjin C.; Ji R.; Ma Y.; Yu X. Microplastics in Agricultural Soils: Sources, Effects, and Their Fate. Curr. Opin Environ. Sci. Health 2022, 25, 100311 10.1016/j.coesh.2021.100311.

[ref72] Andreasi Bassi S.; Boldrin A.; Faraca G.; Astrup T. F. Extended Producer Responsibility: How to Unlock the Environmental and Economic Potential of Plastic Packaging Waste ?. Resour Conserv Recycl 2020, 162, 105030 10.1016/j.resconrec.2020.105030.

[ref73] Stoerk T.; Dudek D. J.; Yang J. China ’ s National Carbon Emissions Trading Scheme: Lessons from the Pilot Emission Trading Schemes, Academic Literature, and Known Policy Details. Climate Policy 2019, 19 (4), 472–486. 10.1080/14693062.2019.1568959.

[ref74] Burdack D.; Biewald A.; Lotze-Campen H. Cap-and-Trade of Water Rights. A Sustainable Way out of Australia’s Rural Water Problems?. GAIA - Ecological Perspectives for Science and Society 2014, 23 (4), 318–326. 10.14512/gaia.23.4.7.

[ref75] Klaassen G.; Lefevere J.; Meadows A.; Runge-metzger A.; Slingenberg Y.; Vergote S.; Werksman J.; Zapfel P.; Vis P. EU Climate Policy; Delbeke J., Vis P., Eds.; Routledge, 2015.

[ref76] Nishida Y.; Hua Y. Motivating Stakeholders to Deliver Change: Tokyo ’ s Cap-and-Trade Program. Building Research & Information 2011, 39 (5), 518–533. 10.1080/09613218.2011.596419.

[ref77] Nielsen M.; Andersen P.; Asche F.; Ellefsen H.; Hammarlund C.; Hoff A.; Kristofersson D. M.; Nielsen R.; Rogvi H. a; Roll K.; Sævaldsson H.; Virtanen J.; Waldo S. Can Small- scale Fisheries Survive Market- Based Management ? Nordic Evidence. Fish and Fisheries 2022, 23 (1), 256–272. 10.1111/faf.12614.

[ref78] Ryberg M. W.; Hauschild M. Z.; Wang F.; Averous-monnery S.; Laurent A. Global Environmental Losses of Plastics across Their Value Chains. Resour Conserv Recycl 2019, 151, 104459 10.1016/j.resconrec.2019.104459.

[ref79] Bauer F.; Holmberg K.; Olsen T.; Stripple J.; Tilsted J. P. Limits to Plastic Growth: Towards a Global Cap on Primary Plastics Production; 2024.

[ref80] Palm E.; Nilsson L. J.; Åhman M. Electricity-Based Plastics and Their Potential Demand for Electricity and Carbon Dioxide. J. Clean Prod 2016, 129, 548–555. 10.1016/j.jclepro.2016.03.158.

[ref81] Abejón R.; Bala A.; Vázquez-rowe I.; Aldaco R.; Fullana-i-palmer P. Resources, Conservation & Recycling When Plastic Packaging Should Be Preferred: Life Cycle Analysis of Packages for Fruit and Vegetable Distribution in the Spanish Peninsular Market. Resour Conserv Recycl 2020, 155, 104666 10.1016/j.resconrec.2019.104666.

[ref82] Ercin A. E.; Aldaya M. M.; Hoekstra A. Y. Corporate Water Footprint Accounting and Impact Assessment: The Case of the Water Footprint of a Sugar-Containing Carbonated Beverage. Water Resources Management 2011, 25 (2), 721–741. 10.1007/s11269-010-9723-8.

[ref83] Stavins R. A U.S. Cap-and-Trade System to Address Global Climate Change. SSRN Journal 2007, 10.2139/ssrn.1026353.

[ref84] Deme G. G.; Ewusi-Mensah D.; Olagbaju O. A.; Okeke E. S.; Okoye C. O.; Odii E. C.; Ejeromedoghene O.; Igun E.; Onyekwere J. O.; Oderinde O. K.; Sanganyado E. Macro Problems from Microplastics: Toward a Sustainable Policy Framework for Managing Microplastic Waste in Africa. Science of the total environment 2022, 804, 150170 10.1016/j.scitotenv.2021.150170. 34517317

[ref85] Simon N.; Raubenheimer K.; Urho N.; Unger S.; Azoulay D.; Farrelly T.; Sousa J.; van Asselt H.; Carlini G.; Sekomo C.; Schulte M. L.; Busch P.-O.; Wienrich N.; Weiand L. A Binding Global Agreement to Address the Life Cycle of Plastics. Science (1979) 2021, 373 (6550), 43–47. 10.1126/science.abi9010. 34210873

[ref86] Farber D. A. Pollution Markets and Social Equity: Analyzing the Fairness of Cap and Trade. Ecol Law Q 2012, 39 (1), 1–56.

[ref87] Welton S. Neutralizing the Atmosphere. Yale Law Journal 2022, 171.

