# Supplementary material for: Opportunities and Challenges of a Cap-and-Trade System for Plastics
Source: Environ Sci Technol. 2025 Jan 24;59(4):1887–96. doi: 10.1021/acs.est.4c04931 (PMC11800383; doi:10.1021/acs.est.4c04931)
Supplement: Supplementary file 1 — es4c04931_si_001.pdf [file es4c04931_si_001.pdf]

Supporting information to:
Opportunities and challenges of a cap-and-trade system for plastics

Hadeel Al-Zawaidah[1*], Marlene Kammerer[2,3], Denise M. Mitrano[4], Kryss Waldschläger[1]

[1]: Wageningen University and Research, Hydrology and Environmental Hydraulics Group, 6700 AA Wageningen, The Netherlands

[2]: Oeschger Centre for Climate Change Research, Institute for Political Science, University of Bern, Fabrikstrasse 8, 3012 Bern, Switzerland

[3]: Department Environmental Social Sciences, eawag Aquatic Research, Überlandstrasse 133, 8600 Dübendorf, Switzerland

[4]: Environmental Systems Science Department, ETH Zurich, Universitätsstrasse 16, 8092 Zurich, Switzerland

[*]Email: hadeel.alzawaidah@wur.nl

Summary of Supporting Information Contents:

- Number of pages: 6
- Number of figures: 0
- Number of tables: 2

Table of content

S1) general review of CAT in different fields S2

S2) grouped lessons from previous CAT in different fields S4

S1) general review of CAT in different fields

Table S1: summary of the lessons learned from different cap and trade systems in different sectors

Sector	Sector-specific anticipated benefits	Sector-specific design features	Sector-specific challenges	Examples	Observations
**Air pollution control**	Flexibility and economic efficiencies that lead to better emissions reduction results while minimizing compliance costs (Stavins 2007; Betsill and Hoffmann 2011)	1. avoidance of prior approvals for trading to reduce transaction costs and improve trading,<br>2. **cap is below business-as-usual (BAU)** conditions to ensure market robustness,<br>3. definition of market rules and availability of accurate emissions data prior to the first compliance period of the system are critical to avoid fluctuations in market prices,<br>4. penalties for non-compliance combined with close monitoring of emissions ensure a high level of compliance,<br>5. banking rules are important for maximizing trading profits and avoiding collapses and price spikes,<br>6. despite the reduced certainty of emissions reductions, price calls (i.e., combining an allowance reserve with the auction price floor) are essential to reduce market volatility and create an investment planning environment by making prices more stable; and<br>7. The use of an **economy-wide system is feasible** (Schmalensee and Stavins 2017)<br>8. The design of an emission trading scheme involves ten design steps, namely; scope definitions, setting the cap, allowance distribution, considering offsets, setting the temporal flexibility, assessing price predictability, sustaining compliance and oversight, capacity building and stakeholders involvement, linkages consideration, continuous feedback and improvement. (PMR and ICAP 2016)	- The perspective adopted in framing the climate change problem and orientation of the solution could underestimate the scope and depth of the climate change problem. (Rosenbloom et al. 2020)<br>- The properties embedded within the policy towards minimizing the economy-wide costs of capping the emission imply a potential interruption to the targeted change. (Rosenbloom et al. 2020)<br>- The approach adopted to support innovation could result in an unmeasurable and arbitrary innovation in the sector towards reduced emissions. (Rosenbloom et al. 2020)<br>- The rigidity of the contextual considerations could reduce the flexibility and adaptation of the policy for sectoral and local contexts. (Rosenbloom et al. 2020)<br>- The political realities can hinder the policy's implementation and anticipated outcome. (Rosenbloom et al. 2020)<br>- The leakage problems are addressed as a defect bound to the CAT policy (Caron et al. 2015), which can be explained by the limited coverage range of the policy to less than 21% (The World Bank 2022).<br>- The divergence between theory and practice is promoted by i) the political restrictions reducing the feasibility of CAT (e.g., Green (2021) and Rafaty et al. (2021)), and ii) the patched and altered nature of CAT in practice as a CAT has not been in place from the beginning (Hahn 1989).	Lead scheme	The success of the scheme is mainly due to two key factors (Hahn 1989):<br>- The existing regulatory apparatus was capable of monitoring lead in gasoline with ease.<br>- An agreement among stakeholders about the primary environmental goals was made prior to the program implementation
				EU emissions trading scheme	- The absence of good data about emissions and the overestimation of allowances relative to actual emissions in 2005 led to the volatility of the EU emissions trading system and the collapse of the market altogether in 2007 (Schmalensee and Stavins 2017).<br>- The experience with the EU trading scheme has shown that key market players can erode the carbon pricing stringency by employing their influential weights (Wettestad 2009).<br>- The involvement of banks has reduced the impact of the temporary market shocks and can help Smooth the variation between supply and demand over time (Hintermann et al. 2016)
				Greenhouse Gas Initiative (RGGI) in the United States	- As the special coverage of the CAT didn't include all the states many industries, migrated to unregulated states, leading to the leakage of carbon emissions in the unregulated states (Yan 2021).
				Carbon emissions in the city of Tokyo	- A mandatory reporting program, where data is shared to investigate the market norms and assess their effectiveness, can help establish a thriving market. (Nishida and Hua 2011)
				GHG emission scheme in China	- The policy development phase included a period for the experimentation and assessment of policy through internal learning (i.e., the process of education on improving policy opinions). This period can motivate a decision-making process that is deliberately and continuously supported by an improved knowledge base (Heggelund et al. 2022).
				The Acid Rain Program	- The success of the scheme was attributed to the sound regulation capacity and the reduced uncertainty for the stakeholders interested in market participation (Borghesi 2014).
**Fisheries**	Management of conflicts between users (Squires et al. 1995) and reduction of uncertainties associated with fish stocks (Sanchirico et al. 2006).	1. cooperative structures, flexible internal transfers, and intertemporal banking are important design features for improving CAT performance (Call and Lew 2015).<br>2. special arrangements (e.g., rules restricting trade in fishing rights among different vessel groups and regions) can provide protection and survival for small-scale fisheries in the context of market reforms (Nielsen et al. 2022).<br>3. trading of fishing permits should be allowed based on the objectives sought (environmental, social, and economic) and the management approach (Serre 2008).	- As fisheries are an economy of scale (Nielsen et al. 2022), CAT is usually associated with a fear of losing small-scale fishing activities, leading to devastating impacts on the coastal communities (Olson 2011; Young et al. 2018; Iversen et al. 2020).<br>- Allocation conflicts between the recreational and commercial sectors are highly probable (Harrison 2021).	The Netherland fisheries permits between 1976 and 1985	- Historical experience in the Netherlands has shown that permit trading can occur even if it is not allowed through legal markets (Smith 2001).
				The pacific habitat in Alaska	- This scheme stands out as a strong example of allocation conflicts between the recreational and commercial sectors (Harrison 2021). Avoiding such conflicts has been seen as possible, however, through case-specific policy arrangements.
**Water pollutants**	Regulation of nonpoint sources (Faeth 2000; King 2005; Collentine 2006).	1. performance of the system is determined by: Restrictiveness of trade rules, information availability, market competition, degree of uncertainty among actors, persistence of transactional relationships among market participants, public support, and direct participation (Borghesi 2014; Woodward et al. 2002).<br>2. governments play a critical role through continuous monitoring and policy enforcement (Woodward et al. 2002).<br>3. nonpoint source pollutants create heterogeneous markets, which require the following: a large proportion of nonpoint source pollutants, costs of nonpoint source pollutant reduction are effective and feasible, and costs of point source pollutant reduction (per unit) are cheaper than nonpoint source pollutant reduction (Zhang and Wang 2002).	- The difficulty in identifying the nonpoint sources of pollutants and the associated difficulty in determining property rights alongside transaction costs (Collentine 2006).	The Lake Taupo Nitrogen Trading Programme (LTNTP)	- The scheme is based on a software that calculates the nitrogen discharge shares for farmers based on different metrics, which lead to a need for continuous update and maintenance to account for the variations of the model parameters over time (e.g., new research findings and land use variation). In the absence of affordable and rapid model updates it can become a barrier in face of trading norms within the market (Spicer et al. 2021).

**Water resources**	Tradable water permits offer better performance compared to taxes and voluntary management practices regarding environmental effectiveness (Young and Karkoski 2000) and/or welfare (Johansson and Moledina 2005).	To establish trading markets, it is necessary to:<br>1. Establish limits on available resources along sustainable lines that are monitored and enforced regularly, and based on science,<br>2. establish property rights that include shares in the consumption pool and access rights, and grant them to users,<br>3. tradability of water shares at low transaction costs and with access and exit restrictions.<br>4. consideration of external costs in shares and allocation prices. (Wheeler et al. 2016)	- Market efficiency is bound to concerns about the monitoring and regulating capacity of the authorities managing the basin encountered by the trading scheme, which includes establishing the cap needed for ecological objectives and issuing sanctions in cases of incompliance (Woodward et al. 2002; Dai et al. 2008; Fisher-Vanden and Olmstead 2013).<br>- As water is an "un-cooperative" commodity, water trading remains challenging (Bakker 2007), where the change in water-use location, timing, and technical efficiency can occur due to trading (Bauer 2004; Young and McColl 2009)<br>- The variety of water resource characteristics can lead to source-specific challenges. To date, CAT adoption for groundwater remains limited compared to surface water (DELWP 2015)<br>- Due to the additional costs bound to water markets (e.g., costs bound to the enforcement of water rights and trading costs) (Garrick et al. 2013), CAT can influence the value of water.<br>- When tradable permits are applied for water resource allocation, concerns about socio-economic aspects associated with water use arise (Meinzen-Dick 2007). The low-income population is expected to suffer more significantly than higher-income populations due to the increase in water prices (Groom et al. 2008; Ruijs et al. 2008)<br>- legal conditions are needed explicitly for water trading to create a separation between water and land ownership (Endo et al. 2018).<br>- Collusions can occur between the holders of the most significant shares of the market, which can lead to limitations in the shift towards environmentally friendly alternatives and technology due to permit price limitations (Hahn and Hester. 1989; Hagem and Westskog 1998)	The San Joaquin Basin markets	- 56% of the total regional allocation was controlled by one person managing two out of eight participants in the market, resulting in serious market competition limitations issues (Young and Karkoski 2000).
				Water market experience in China	- The market development is challenged by the lack of trust and commitment accompanied by the difficulties in integrating water markets with other policies in place to tackle water scarcity and<br>- The distinctive contextual feature of the different pilots implemented limit their applicability on a larger scale and within other contexts. (Moore 2015)
				water management in the Hangjin district, China	- When volumetric water rights systems are employed in replacement of traditional water allocations for irrigation (i.e., managing the order and length of the water extraction period among the involved parties) are still working they could lead to conflicts.<br>- While volumetric-based allocation requires an announcement of the shares by the regulating authorities, a time-based resource allocation can reduce the administrative cost reduce conflicts with traditional management methods. (Zheng et al. 2012)
				Water allocation scheme in Australia (mainly the Murray-Darling Basin)	- the Australian government's financial capacity to mitigate against over-allocation by buying back permits has improved compliance with the anticipated environmental targets (Burdack et al. 2014).

S2) grouped lessons from previous CAT in different fields

Table S2: summary of the lessons learned from different cap and trade systems in different sectors, summarized based on the three key design features

Contextual features	Water resources	Water pollutants	Fisheries	Air pollution control
Cap establishment and permits allocation	• It is crucial to establish limits on available resources (Wheeler et al. (2016)) The shares held per market player should be low enough to insure market robustness and avoid monopolies formation , resulting in serious market competition limitations issues (Young and Karkoski 2000).<br>• The allocation of permits should be done taking into account the traditional and costumery norms within CAT boundary (Zheng et al. 2012)<br>• To establish trading markets, it is necessary to:<br>1. Establish property rights that include shares in the consumption pool and access rights, and grant them to users,<br>• Account for external costs in shares and allocation prices. (Wheeler et al. (2016).)<br>• Collusions between the holders of the most significant shares of the market should be avoided as it can hinder the shift towards environmentally friendly alternatives and technology (Hahn and Hester. 1989; Hagem and Westskog 1998)<br>• Bbuilding trust and commitment from the primary stakeholders are key factors for market development (Moore 2015)	• Software can be used to calculate shares for involved stakeholders. In the absence of affordable and rapid model updates it can become a barrier in face of trading norms within the market (Spicer et al. 2021).-	• Conflicts between different sectors under the cap should be considered within the permits phase (Harrison 2021).-	• The set cap should ensure market robustness, therefore the cap should be below business-as-usual (BAU) (Schmalensee and Stavins 2017)<br>• An economy-wide cap system is feasible, covering the full temporal scale of air pollutants (Schmalensee and Stavins 2017)<br>• Insuring a cap covering the full spatiotemporal scale of air pollutants is crucial (Yan 2021).<br>• data availability is crucial for sound estimation of allowances is necessary to avoid market volatility and collapse (Schmalensee and Stavins 2017).<br>• An agreement between market players about the primary environmental goals should be established prior to the program implementation (Hahn 1989)<br>•
Establishment of a fostering environment of the market<br>,	• Shares should be tradable at low transaction costs and with flexible access and exit restrictions (Wheeler et al. (2016).<br>• Market efficiency is determined by the monitoring and regulating capacity of the authorities managing the trading scheme, which includes establishing the cap needed for ecological objectives and issuing sanctions in cases of incompliance (Woodward et al. 2002; Dai et al. 2008; Fisher-Vanden and Olmstead 2013).<br>• Due to the additional costs bound to markets (e.g., costs bound to the enforcement of permits trading costs) (Garrick et al. 2013), CAT can influence the value of resource/ pollutant encountered under the policy.<br>• Concerns about socio-economic aspects arise (Meinzen-Dick 2007). The low-income population is expected to suffer more significantly than higher-income populations due to the build-up of the associated cost of the end product (Groom et al. 2008; Ruijs et al. 2008)<br>• The government's financial capacity to mitigate against over-allocation by buying back permits has improved compliance with the anticipated environmental targets (Burdack et al. 2014).	• Performance of the system is determined by: Restrictiveness of trade rules, information availability, market competition, uncertainty, persistence of transactional relationships among market participants, public support, and direct participation (Borghesi 2014; Woodward et al. 2002).<br>• Governments play a critical role through continuous monitoring and policy enforcement (Woodward et al. 2002).	• Cooperative structures, flexible internal transfers, and intertemporal banking are important design features for improving CAT performance (Call and Lew 2015).<br>• Trading of permits should be allowed based on the objectives sought (environmental, social, and economic) and the management approach (Serre 2008).<br>• Historical experience has shown that permit trading can occur even if it is not allowed through legal markets (Smith 2001).<br>• Special arrangements (e.g., rules restricting trade in fishing rights among different vessel groups and regions) can provide protection and survival for small-scale businesses in the context of market reforms (Nielsen et al. 2022).<br>• For economy of scale (Nielsen et al. 2022), CAT is could lead to losing small-scale activities, leading to devastating impact on the involved communities (Olson 2011; Young et al. 2018; Iversen et al. 2020).	• Definition of market rules and availability of accurate emissions data prior to the first compliance period of the system are critical to avoid fluctuations in market prices<br>• Avoidance of prior approvals for trading to reduce transaction costs and improve trading<br>• Penalties for non-compliance combined with close monitoring of emissions ensure a high level of compliance,<br>• Banking rules are important for maximizing trading profits and avoiding collapses and price spikes,<br>• Price calls (i.e., combining an allowance reserve with the auction price floor) are essential to reduce market volatility and create an investment planning environment by making prices more stable (Schmalensee and Stavins 2017)<br>• Sound regulation capacity and reduced uncertainty for the stakeholders increases market participation and the policy performance all together (Borghesi 2014).<br>• The cap should cover the full special scale of the market to minimize leakages ((Caron et al. 2015), (The World Bank 2022), and (Yan 2021)).<br>• Innovation should be supported under measurable criteria towards supporting achieving the targeted VAT (Rosenbloom et al. 2020)<br>• The influential weights of the key market player should not be overlooked as it can erode the policy stringency (Wettestad 2009).
	• The market development is challenged by difficulties in integrating markets with other polices in place tackling environmental challenges (Moore 2015)<br>• The market output is highly dependent on the distinctive feature of the scheme, challenging a unified larger scale and within other contexts. (Moore 2015)<br>• The development of the scientific background and continuous feedback about the market and cap performance should guide the policy development and adaptation over its life span (Wheeler et al. (2016))	-	-	• The policy should be flexible and adaptable to sectoral and local contexts with minimal contextual considerations rigidity (Rosenbloom et al. 2020).<br>• The political realities should be acknowledged and incorporated within the policy design. (Rosenbloom et al. 2020).<br>• Internal learning through pilots studies where multiple policy design models are explored can improve the final policy design (Heggelund et al. 2022).<br>• The divergence between theory and application should be minimised through mitigation against political restrictions on the CAT feasibility (e.g., Green (2021) and Rafaty et al. (2021)), which could limit the effectiveness of CAT<br>• Reporting program should be mandatory as it supports the establishment of thriving market (Nishida and Hua 2011)
Material and targeted group Identification	• As water is an "un-cooperative" commodity, water trading remains challenging (Bakker 2007), where the change in water-use location, timing, and technical efficiency can occur due to trading (Bauer 2004; Young and McColl 2009)<br>• The variety of resource characteristics can lead to source-specific challenges (DELWP 2015)	• The diversity of resources increases the difficulty in identifying the nonpoint sources of pollutants and the needed property rights alongside transaction costs (Collentine 2006).	-	-

References:

Bakker K (2007) The " Commons " Versus the Anti-privatization and the Human Right to Water in the Global South. Antipode 39:430–455

Bauer CJ (2004) Results of Chilean water markets: Empirical research since 1990. Water Resour Res 40:1–11. https://doi.org/10.1029/2003WR002838

Betsill M, Hoffmann MJ (2011) The Contours of "Cap and Trade": The Evolution of Emissions Trading Systems for Greenhouse Gases. 28:83–106

Borghesi S (2014) Water tradable permits : a review of theoretical and case studies. Journal of Environmental Planning and Management 57:1305–1332. https://doi.org/10.1080/09640568.2013.820175

Burdack D, Biewald A, Lotze-Campen H (2014) Cap-and-trade of Water Rights. A Sustainable Way out of Australia's Rural Water Problems? GAIA - Ecological Perspectives for Science and Society 23:318–326. https://doi.org/10.14512/gaia.23.4.7

Caron J, Rausch S, Winchester N (2015) Leakage from sub-national climate policy: The case of California's cap-and-trade program. The Energy Journal 36:167–190. https://doi.org/10.5547/01956574.36.2.8

Collentine D (2006) Composite market design for a Transferable Discharge Permit (TDP) system. Journal of Environmental Planning and Management 49:929–946. https://doi.org/10.1080/09640560600947055

Dai TS, Gu BY, Zhao WH (2008) Study on the market power in water rights market. In: 2008 4th International Conference on Wireless Communications, Networking and Mobile Computing. IEEE, pp 1–5

DELWP (2015) Victorian Water Trading 2014–15 Annual Report

Endo T, Kakinuma K, Yoshikawa S, Kanae S (2018) Are water markets globally applicable? Environmental Research Letters 13:034032. https://doi.org/10.1088/1748-9326/aaac08

Faeth P (2000) Fertile Ground: Nutrient Trading's Potential to Cost-Effectively Improve Water Quality. World Resources Institute, Washington, DC

Fisher-Vanden K, Olmstead S (2013) Moving Pollution Trading from Air to Water: Potential, Problems, and Prognosis. Journal of Economic Perspectives 27:147–172. https://doi.org/10.1257/jep.27.1.147

Garrick D, Whitten SM, Coggan A (2013) Understanding the evolution and performance of water markets and allocation policy: A transaction costs analysis framework. Ecological Economics 88:195–205. https://doi.org/10.1016/j.ecolecon.2012.12.010

Green JF (2021) Does carbon pricing reduce emissions? A review of ex-post analyses. Environmental Research Letters 16:043004. https://doi.org/10.1088/1748-9326/abdae9

Groom B, Liu X, Swanson T, Zhang S (2008) Resource pricing and poverty alleviation: The case of block tariffs for water in Beijing. In: Koundouri P (ed) Coping with water deficiency. Springe, Dordrecht, pp 213–237

Hagem C, Westskog H (1998) The Design of a Dynamic Tradeable Quota System under market imperfections. J Environ Econ Manage 36:89–107

Hahn RW (1989) Economic Prescriptions for Environmental Problems: How the Patient Followed the Doctor's Orders. Journal of Economic Perspective 3:95–114

Hahn RW, Hester. GL (1989) Marketable Permits: Lessons From Theory and Practice. Ecol Law Q 16:361–406

Harrison HL (2021) Managing many nets: Possible scenarios and impacts for the expansion of Cook Inlet personal use fisheries. Fish Res 236:105811. https://doi.org/10.1016/j.fishres.2020.105811

Heggelund G, Stensdal I, Duan M (2022) China's Carbon Market: Potential for Success? Politics and Governance 10:265–274. https://doi.org/10.17645/pag.v10i1.4792

Hintermann B, Peterson S, Rickels W (2016) Price and market Behavior in phase II of the EU ETS: A review of the literature. Rev Environ Econ Policy 10:108–128. https://doi.org/10.1093/reep/rev015

Iversen A, Asche F, Buck M, et al (2020) The growth and decline of fisheries communities : Explaining relative population growth at municipality level. Mar Policy 112:103776. https://doi.org/10.1016/j.marpol.2019.103776

Johansson RC, Moledina AA (2005) Comparing Policies to Improve Water Quality when Dischargers of Pollutants are Strategic. Water Int 30:166–173. https://doi.org/10.1080/02508060508691857

King DM (2005) Crunch time for water quality trading. Choices 20:71–75

Meinzen-Dick R (2007) Beyond panaceas in water institutions. Proceedings of the National Academy of Sciences 104:15200–15205. https://doi.org/10.1073/pnas.0702296104

Moore SM (2015) The development of water markets in China : progress , peril , and prospects. Water Policy 17:253–267. https://doi.org/10.2166/wp.2014.063

Nielsen M, Andersen P, Asche F, et al (2022) Can small- scale fisheries survive market- based management ? Nordic evidence. Fish and Fisheries 23:256–272. https://doi.org/10.1111/faf.12614

Nishida Y, Hua Y (2011) Motivating stakeholders to deliver change : Tokyo ' s Cap-and-Trade Program. Building Research & Information 39:518–533. https://doi.org/10.1080/09613218.2011.596419

Olson J (2011) Ocean & Coastal Management Understanding and contextualizing social impacts from the privatization of fi sheries : An overview. Ocean Coast Manag 54:353–363. https://doi.org/10.1016/j.ocecoaman.2011.02.002

PMR, ICAP (2016) Emissions trading in practice: A handbook on design and implementation

Rafaty R, Dolphin G, Pretis F (2021) Carbon Pricing and the Elasticity of CO_2 Emissions

Rosenbloom D, Markard J, Geels FW, Fuenfschilling L (2020) Why carbon pricing is not sufficient to mitigate climate change—and how "sustainability transition policy" can help. Proceedings of the National Academy of Sciences 117:8664–8668. https://doi.org/10.1073/pnas.2004093117

Ruijs A, Zimmermann A, Van Den Berg M (2008) Demand and distributional effects of water pricing policies. Ecological Economics 66:506–516. https://doi.org/10.1016/j.ecolecon.2007.10.015

Sanchirico JN, Holland D, Quigley K, Fina M (2006) Catch-quota balancing in multispecies individual fishing quotas. Mar Policy 30:767–785. https://doi.org/10.1016/j.marpol.2006.02.002

Schmalensee R, Stavins RN (2017) Lessons Learned from Three Decades of Experience with Cap and Trade. Rev Environ Econ Policy 11:59–79. https://doi.org/10.1093/reep/rew017

Smith W (2001) Dutch demersal North Sea fisheries initial allocation of flatfish ITQs. FAO FISHERIES TECHNICAL PAPER 15–23

Spicer EA, Swaffield S, Moore K (2021) Agricultural land use management responses to a cap and trade regime for water quality in Lake Taupo catchment, New Zealand. Land use policy 102:105200. https://doi.org/10.1016/j.landusepol.2020.105200

Squires D, Kirkley J, Tisdell CA (1995) Individual transferable quotas as a fisheries management tool. Reviews in Fisheries Science 3:141–169. https://doi.org/10.1080/10641269509388570

Stavins R (2007) A U.S. cap-and-trade system to address global climate change (Hamilton Project Discussion Paper 2007-13)

The World Bank (2022) Carbon pricing dashboard [Dataset]. https://carbonpricingdashboard.worldbank.org/map_data. Accessed 30 May 2022

Wettestad J (2009) EU Energy-Intensive Industries and Emission Trading: Losers Becoming Winners? Environmental Policy and Governance 19:309–320. https://doi.org/10.1002/eet.516

Wheeler SA, Schoengold KS, Bjornlund H (2016) Lessons to be learned from groundwater trading in Australia and the United States. In: In Integrated groundwater management. Springer, Cham, pp 493–517

Woodward RT, Kaiser RA, Wicks AB (2002) THE STRUCTURE AND PRACTICE OF WATER QUALITY TRADING MARKETS. JAWRA Journal of the American Water Resources Association 38:967–979

Yan J (2021) The impact of climate policy on fossil fuel consumption : Evidence from the Regional Greenhouse Gas Initiative (RGGI). Energy Econ 100:105333. https://doi.org/10.1016/j.eneco.2021.105333

Young MD, McColl JC (2009) Double trouble: the importance of accounting for and defining water entitlements consistent with hydrological realities. Australian Journal of Agricultural and Resource Economics 53:19–35. https://doi.org/10.1111/j.1467-8489.2007.00422.x

Young OR, Websterb DG, Cox ME, et al (2018) Moving beyond panaceas in fisheries governance. In: Proceedings of the National Academy of Sciences. pp 9065–9073

Young TF, Karkoski J (2000) Green evolution are economic incentives the next step in nonpoint source pollution control. Water Policy 2:151–173

Zheng H, Wang Z, Calow R, Wei Y (2012) Water Rights Allocation, Management and Trading in an Irrigation District - A Case Study of Northwestern China. In: Kumar M (ed) Problems, Perspectives and Challenges of Agricultural Water Management. InTech
